# Intraosseous Lidocaine Trigger Point Injection for Vertebrogenic Pain: From Molecular Principles to Clinical Applications: A Narrative Review

**DOI:** 10.3390/ijms27198935

**Published:** 2026-10-08

**Authors:** Mustafa Al-Zamil, Natalia A. Shnayder, Marina M. Petrova, Margarita V. Naprienko, Ivan V. Zhelambekov, Olga V. Goncharova, Natalia P. Garganeeva, Svetlana V. Kuleshova, Oxana V. Eremina, Anna O. Dobrosmyslova

**Affiliations:** 1 Department of Physiotherapy, Faculty of Continuing Medical Education, Peoples’ Friendship University of Russia, 117198 Moscow, Russia; 2 Institute of Personalized Psychiatry and Neurology, V.M. Bekhterev National Medical Research Center for Psychiatry and Neurology, 3 Bekhterev St., 192019 St. Petersburg, Russia; 3 Shared Core Facilities “Molecular and Cell Technologies”, V.F. Voino-Yasenetsky Krasnoyarsk State Medical University, 1 Partizan Zheleznyak St., 660022 Krasnoyarsk, Russia; stk99@yandex.ru; 4 Department of Sports Medicine and Medical Rehabilitation, Sechenov First Moscow State Medical University, 119991 Moscow, Russia; mv_naprienko@mail.ru (M.V.N.); olga.goncharova014@gmail.com (O.V.G.); 5 Department of Restorative Medicine and Neurorehabilitation, Medical Dental Institute, 127253 Moscow, Russia; aiybolit69@mail.ru; 6 Department of General Medical Practice and Outpatient Therapy, Siberian State Medical University, 634050 Tomsk, Russia; garganeeva@gmail.com; 7 Federal State Budgetary Institution “United Hospital with a Polyclinic” of the Executive Office of the President of the Russian Federation, 119330 Moscow, Russia; 8 Department of Cardiology, Functional and Clinical Laboratory Diagnostics, Institute of Postgraduate Education, V.F. Voino-Yasenetsky Krasnoyarsk State Medical University, 1 Partizan Zheleznyak Str., 660022 Krasnoyarsk, Russia; eryomina@mail.ru; 9 Federal Siberian Research and Clinical Center of the Federal Medical and Biological Agency, 26 Kolomenskaya St., 660037 Krasnoyarsk, Russia; a-dobrosmyslova@mail.ru

**Keywords:** intraosseous lidocaine trigger point injection, intraosseous receptors, nociceptive pain, neuropathic pain, nociplastic pain, lidocaine blockade, intraosseous blockade, vertebrogenic pain

## Abstract

Intraosseous lidocaine trigger point injection (LTPI) has been utilized for an extended period in the management of chronic vertebrogenic pain; yet, its molecular mechanisms and clinical efficacy require critical appraisal. This narrative review highlights the milestones of osteoreception and elucidates the molecular mechanisms driving intraosseous receptor irritation and its pivotal role in chronic pain development. Pathological vertebral bone remodeling triggers a receptor activator of nuclear factor-kappa B ligand/osteoprotegerin (RANKL/OPG) imbalance, accelerating hyperactive osteoclastogenesis. Enhanced osteoclastic vacuolar-type H+-ATPase (V-ATPase) expression drives microenvironment acidity and intraosseous hypertension, persistently stimulating acid-sensing ion channel 3 (ASIC3) and purinergic P2X2/3 receptors on intraosseous afferents. Concurrently, upregulated nerve growth factor (NGF) binds to tropomyosin receptor kinase A (TrkA), driving peripheral sensitization via transient receptor potential vanilloid 1 (TRPV1) phosphorylation. The available clinical studies investigating intraosseous LTPI for vertebrogenic pain are scarce and heterogeneous, focusing primarily on low back pain, cervicalgia, and residual chronic pain after degenerative lumbar spinal stenosis decompression surgery. The reviewed data provide preliminary signals of potential benefit, observing a statistically significant analgesic effect of intraosseous LTPI within the studied cohorts. In two trials comparing it to intramuscular LTPI, one of which utilized a randomized controlled design, the intraosseous approach was reported to provide more pronounced short-term pain relief. However, these trials are limited by small sample sizes, heterogeneous designs, and a lack of active long-term safety monitoring. Nevertheless, although current data provide promising but still limited insights, future double-blinded, multicenter randomized controlled trials are required to comprehensively establish the clinical efficacy, safety profile, and prolonged effect of intraosseous LTPI in the treatment of vertebrogenic pain.

## 1. Introduction

Conservative treatment of patients with vertebrogenic pain remains one of the most pressing challenges in modern medicine [1,2,3]. This urgency is driven, on one hand, by the high prevalence of these conditions [4,5], and on the other, by the resulting increase in disability and loss of work capacity due to frequent exacerbations of the pain syndrome [6,7]. Most commonly, clinicians encounter two distinct types of vertebrogenic pain: chronic primary vertebrogenic pain (CPVP) and residual post-decompression lumbar vertebrogenic pain (RLVP).

The CPVP syndrome is a type of chronic primary musculoskeletal pain characterized by persistent pain lasting longer than 3 months in vertebral muscles, bones, joints, or tendons. While vertebrogenic pain is directly associated with intraosseous changes in the vertebral column and alterations in the vertebral endplates [8], this diagnosis strictly excludes deep somatic lesions, including systemic pathologies, rheumatic disorders, oncological diseases, inflammation, or the biomechanical consequences of nervous system disorders [9]. The role of intraosseous nociceptors in the development of CPVP has been demonstrated in numerous clinical studies [10,11,12,13]. According to Dr. Modic, bone tissue changes in spondylosis are classified based on MRI findings into three groups: type 1 represents acute inflammation and edema with low T1 and high T2 signals (Modic 1); type 2 indicates fatty infiltration of the bone marrow with high T1 and high T2 signals (Modic 2); and type 3 denotes subchondral bone sclerosis with low T1 and low T2 signals (Modic 3) [14].

In contrast, RLVP is recognized as a specific clinical entity, frequently characterized by intractable low back pain, often accompanied by radiating leg pain. The current literature indicates that the prevalence of this pain syndrome reaches 30.3% among patients who have undergone degenerative lumbar spinal stenosis decompression surgery [15,16]. The clinical significance of RLVP lies in its profound resistance to standard pharmacotherapy and physical therapy, the absence of clear indications for revision spinal surgery in the majority of cases, and a high morbidity rate of 23.7% associated with recurrent interventions [17].

Given the multifactorial degenerative changes associated with these diseases, the pain syndrome typically consists of three distinct components. The nociceptive component arises from afferentation due to structural tissue damage. In parallel, the neuropathic component is driven by nerve fiber injury and subsequent peripheral and central sensitization. Crucially, growing evidence supports the involvement of the nociplastic component, which results from an overactive or dysfunctional central nociceptive system that does not correlate with the influx of nociceptive or neuropathic afferentation [18,19,20].

Specifically, these two pathological conditions, where surgical intervention is either unnecessary or ineffective, present significant challenges for clinicians selecting an appropriate therapeutic strategy during acute exacerbations. This is particularly evident when the pain syndrome is highly pronounced and accompanied by antalgic postures and pain-induced immobility [21]. The administration of standard pharmacological therapy, combined with non-pharmacological interventions such as physiotherapeutic modalities, manual therapy, and acupuncture, exhibits low efficacy in managing these pathological states in many cases [22,23,24]. As a result, conservative approaches fall short of meeting the urgent clinical requirement for rapid, albeit temporary, pain control. Patients experiencing such debilitating conditions can neither tolerate protracted therapies nor afford the time required for prolonged rest, restorative sleep, and recovery from persistent fatigue [25,26]. These interconnected disturbances establish a vicious cycle that progressively exacerbates pain severity and impedes the rehabilitation process [27]. Under these constraints, therapeutic blockade constitutes an essential therapeutic option.

Although intramuscular trigger point injections are established as a highly effective approach for severe pain management [28,29], their therapeutic efficacy remains limited in many patients with resistant pain syndromes. To overcome these limitations, an alternative approach targeting the blockade of intraosseous receptors has been progressively developed since 1985 [30]. This procedure is variously known as intraosseous blockade, bone blockade, lidocaine bone blockade, or Sokov’s blockade. However, all these terms are unified in accordance with the international nomenclature as intraosseous LTPI. The integration of these diverse terms under the comprehensive designation of Intraosseous Lidocaine Trigger Point Injection (LTPI) is both scientifically justified and logical, as it precisely reflects three fundamental principles shared by all these modalities: the anatomical approach (intraosseous), the target site (trigger point), and the primary therapeutic agent (lidocaine) [15].

To our knowledge, the first application of intraosseous anesthetic injection for the treatment of chronic musculoskeletal pain was conducted by the Russian traumatologist G.M. Shulyak. Notably, the author was the first to investigate the physiological and anatomical foundations of intraosseous procaine (Novocaine) injections, publishing his findings in the 1953 monograph, “Intraosseous Method of Pain Relief and its Anatomical Basis” [15]. At that time, the use of lidocaine for blocks was limited, as it had only recently been synthesized. Consequently, the first published reports on intraosseous lidocaine administration for the treatment of vertebrogenic pain appeared in 1985 in the works of Professor E.L. Sokov, under whose supervision a series of comprehensive clinical studies have been conducted continuously up to the present day [30].

Despite the active clinical use of intraosseous LTPI in Russia and several other countries, the established pathogenetic framework of this method is primarily based on the experimental studies of Latvian neurophysiologist G. Yankovsky. Yankovsky investigated osteoreception by recording evoked potentials across various cerebral structures from the tibial interosseous nerve in response to increased intraosseous pressure induced by fluid injection into cancellous bone [31]. The second cornerstone of this treatment modality is the gate control theory of pain [15].

However, rigorous molecular-level investigations into the analgesic mechanisms of intraosseous LTPI remain critically scarce. Furthermore, existing clinical efficacy studies on intraosseous LTPI have largely stayed in the shadows due to limited publications, a persistent language barrier, and the historical nature of many foundational papers. Given the high clinical relevance of vertebrogenic pain and the potential efficacy of this treatment approach, a modern synthesis is urgently required. To address this gap in scientific knowledge, this narrative review aims to provide a comprehensive synthesis of the pathogenetic, molecular, structural, and neuroanatomical bases of the intraosseous changes underlying vertebrogenic pain, describe the administration techniques, mechanisms of action, and potential complications of intraosseous LTPI, and evaluate the clinical efficacy of intraosseous LTPI in the treatment of CPVP and RLVP.

## 2. Materials and Methods

This study was designed as a structured narrative review aimed at extracting, analyzing, and synthesizing literature data on intraosseous LTPI for chronic vertebrogenic pain. To ensure methodological transparency and minimize selection bias, the review process was guided by a structured literature monitoring framework. The specific protocol for this review was officially registered in the Open Science Framework (OSF) under the DOI: https://doi.org/10.17605/OSF.IO/96PXA (accessed on 1 October 2026).

### 2.1. Eligibility

To balance a comprehensive scope with a focused analysis, distinct eligibility criteria were applied to the different components of the review. The review included studies focusing on the underlying molecular, physiological, histological, and anatomical mechanisms of intraosseous changes in the development of chronic vertebrogenic pain. Additionally, it covered intraosseous LTPI techniques, reported challenges and complications, and clinical studies investigating the applications of intraosseous lidocaine administration for the treatment of chronic vertebrogenic pain. Eligibility was extended to all available literature materials published in the English and Russian languages.

### 2.2. Search Strategy

To identify relevant publications, a comprehensive literature search was performed across major biomedical databases, including Medline/PubMed, Embase, the Cochrane Library, and eLIBRARY.RU, using combinations of free-text keywords, Medical Subject Headings (MeSH), and Emtree terms without chronological restrictions. The search architecture was systematically organized into targeted clusters of terms to capture the entire continuum from basic science to clinical practice, as detailed in Supplementary Figure S1.

### 2.3. Screening

Study screening for the clinical section was executed in two distinct, sequential phases:

- Title and Abstract Screening: Two reviewers (M.V.N. and N.A.S.) independently screened titles and abstracts against the pre-specified eligibility criteria.
- Full-Text Screening: The full texts of potentially eligible studies were retrieved and independently assessed by the same two reviewers. Notably, to eliminate potential authorial bias, any eligibility decisions regarding primary publications co-authored by the screening team were immediately referred to and finalized by independent reviewers (O.V.G. and A.O.D.), as detailed in Section 2.5.

### 2.4. Data Extraction

Data extraction was summarized textually. A formal risk of bias assessment was not performed, which aligns with the standard methodological approach for narrative reviews.

### 2.5. Data Synthesis and Mapping

The synthesized evidence was structured and conceptually mapped across three distinct thematic areas to follow the trajectory from basic science to clinical practice of intraosseous LTPI:

1. Molecular and Pathogenetic Mechanisms of Osteoreception in Chronic Vertebrogenic Pain (performed by M.A.-Z. and M.M.P.).
2. Technique and Therapeutic Pathways of Intraosseous LTPI (performed by S.V.K. and O.V.E.).
3. Clinical applications of intraosseous LTPI for vertebrogenic pain (performed by O.V.G. and A.O.D.).

To ensure complete transparency and eliminate potential authorial bias, eligibility decisions, data-extraction verification, and the formal study-level methodological appraisal for any publications co-authored by the primary investigators (M.A.-Z., N.A.S., M.M.P., and I.V.Z.) were strictly assigned to and independently audited by O.V.G. and A.O.D., who possessed no prior involvement or affiliation with the primary clinical research.

## 3. Results

### 3.1. Molecular and Pathogenetic Mechanisms of Osteoreception in Chronic Vertebrogenic Pain

#### 3.1.1. Neuroanatomical Distribution and Molecular Phenotype of Intraosseous Afferent Fibers

Both Aδ- and C-fiber sensory neurons innervate the bone marrow [32,33,34]. While the periosteum contains an equally high concentration of both types, C-fibers predominate in the cancellous bone [34]. Electrophysiological recordings demonstrate that administering capsaicin into the bone marrow cavity increases the ongoing activity of these bone afferents, particularly the C-fibers, indicating their leading role in intraosseous nociception [35]. Degenerative changes in the cancellous bone trigger an increased expression of TRPV1, ASIC3, and P2X2/3, resembling the hyperinnervation of peripheral terminals [35,36,37]. This tissue-specific difference is also evident in the distribution of autonomic innervation; although sympathetic fibers outnumber sensory ones in the periosteum, their quantity in cancellous bone is not significantly different [34]. It has long been established that calcitonin gene-related peptide (CGRP) and 200-kDa neurofilament (NF200)-positive sensory fibers in outer and inner layers of the periosteum lack a clear association with CD31+ blood vessels. Instead, these nerve fibers form a unique, net-like meshwork within the periosteum, which is heavily involved in detecting both algogenic substances and fracture-induced mechanical distortion of the underlying cortical bone [38]. Approximately 80% of unmyelinated or thinly myelinated sensory nerve fibers expressing calcitonin gene-related peptide (CGRP) that innervate the periosteum, mineralized bone, and bone marrow also express TrkA. Similarly, the majority of myelinated sensory fibers expressing neurofilament 200 kDa (NF200) within these skeletal tissues co-express TrkA. The observation that most skeleton-innervating sensory nerve fibers are TrkA-positive may partially explain why therapies targeting the NGF/TrkA pathway are highly effective in attenuating bone pain [39].

#### 3.1.2. Aging and Osteoclast Differentiation Factors

Numerous bone diseases, particularly those associated with aging, are characterized by the hyperactivation of osteoclasts, the primary cells responsible for bone resorption. Osteoclast differentiation is strictly governed by three interconnected factors: macrophage colony-stimulating factor (M-CSF), receptor activator of nuclear factor-kappa B ligand (RANKL), and osteoprotegerin (OPG) [40,41,42]. According to Chung et al., the in vitro generation of osteoclasts and the constitutive expression of RANKL in enriched marrow stromal cells increase with age, whereas OPG expression is inversely correlated with aging [42]. Lysosomes play a pivotal role in executing osteoclast functions, serving as catabolic hubs that regulate macromolecule degradation, intracellular vesicle trafficking, and autophagy [43]. Morphologically, lysosomes are membrane-bound organelles enclosed by a single phospholipid bilayer and filled with over 60 distinct degradative enzymes (acid hydrolases). Each enzyme targets specific substrates for degradation, which collectively determine the lysosome’s total hydrolytic capacity [44]. Crucially, beyond intracellular digestion, these organelles actively drive extracellular bone remodeling via lysosomal exocytosis, serving as the primary vehicle for delivering acidification machinery to the cell surface.

Lysosomes during bone resorption fuse with the bone-facing plasma membrane of activated osteoclasts (OCs) to form the ruffled border (RB), transforming the isolated resorption lacuna into a functional “giant extracellular lysosome.” Driven by lysosome-associated membrane proteins 1 and 2 (LAMP1 and LAMP2), this tightly orchestrated biogenesis enables the RB to serve as a specialized secretory organelle that pumps protons and proteases directly into the resorption compartment to dissolve the bone matrix. Concurrently, lysosomes act as critical calcium ion Ca^2+^ storage organelles; the efflux of lysosomal Ca^2+^ through channels such as transient receptor potential mucolipin 1 (TRPML1) triggers intracellular Ca^2+^oscillations that activate the nuclear factor of activated T cells 1 (NFATc1), the key transcription factor for osteoclast differentiation. To sustain this high-density bone degradation, chloride channel type 7 (ClC-7) functions as a chloride/proton (Cl^-^/H^+^) antiporter at the RB, maintaining the essential ionic and pH balance required for continuous extracellular acidification [43].

#### 3.1.3. Inflammaging-Related Changes in Intraosseous Nociceptors

Although aging is an inevitable physiological process, it is frequently accompanied by various pathological conditions, including pain and functional impairment [45,46]. The primary molecular changes associated with aging are linked with metabolic stress, mitochondrial dysfunction, and caloric restriction, which trigger a sharp increase in reactive oxygen species, structural DNA damage and protein dysfunction, cellular senescence and stem cell exhaustion, resulting in decreased cellular proliferation, altered cytokine secretion, and cellular apoptosis [47,48,49,50,51,52].

Essentially, these molecular and cellular interactions serve as a key component in the development of chronic musculoskeletal pain. During the aging-associated shift, the dysregulation and subsequent elevation of nerve growth factor (NGF) levels in bone tissue transform this neuropeptide from a homeostatic regulator—which normally modulates immune cell function, influences angiogenesis, and participates in bone remodeling [53]—into a primary driver of intraosseous receptor irritation [54,55].

**Figure 1:**
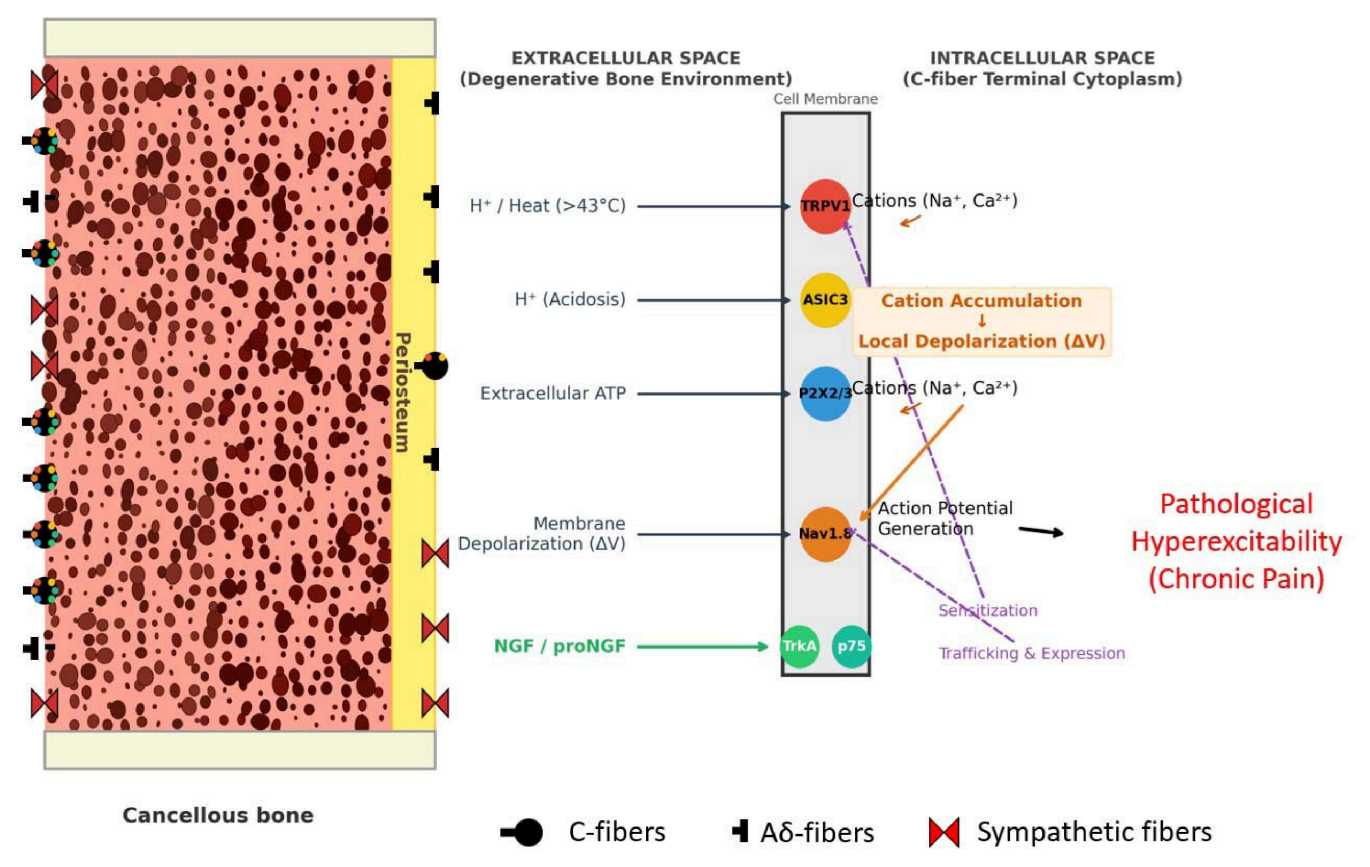
Molecular Drivers of Pathological Bone Pain and Nociceptor Hyperexcitability.

Experimentally, Nencini et al. demonstrated a close link between NGF exposure and the development of bone pain. This conclusion was reached through in vivo rat experiments, which identified specific receptors on the membranes of intraosseous osteoreceptors. Upon binding, NGF rapidly activates bone nociceptors and acutely sensitizes single mechanically activated bone nociceptors [55]. Several types of NGF receptors and associated channels have been distinguished in rat bone tissue, including the p75 neurotrophin receptor, tropomyosin receptor kinase A (TrkA), transient receptor potential vanilloid 1 (TRPV1), and the voltage-gated sodium channel (Na1.8). NGF binds to both the p75 receptor and the TrkA receptor. While the p75 receptor exhibits comparable affinity for all neurotrophins, the TrkA receptor—a member of the homologous Trk receptor family—displays high affinity and strict selectivity for NGF [32]. Furthermore, it has been demonstrated that direct intra-tibial injection of NGF rapidly activates and sensitizes these nociceptors, inducing acute behavioral responses on a similar time course [55]. In contrast, the administration of anti-NGF inhibitors has been introduced to many skeletal diseases for pain control [54] (Figure 1).

#### 3.1.4. Intraosseous Hypertension and Hyperexcitability of Intraosseous Receptors

Intraosseous hypertension is assumed to arise from obstructed venous backpressure or intrinsic tissue turgor, and it is closely associated with bone pain and osteonecrosis [56]. In the healthy population, the mean humeral intraosseous pressure has been detected at a level exceeding the external mean blood pressure by 26.5% ± 15.2% [57]. Values exceeding 40 mmHg are generally considered pathological [58]. However, a three- to five-fold increase above the normal baseline pressure is required to reach the threshold for activating intraosseous mechanoreceptors [55]. Experimentally, the neurophysiological mechanisms linking intraosseous hypertension to pain transmission have been demonstrated in animal models. Ishida et al. (2016) showed that artificial elevation of intraosseous pressure via balloon inflation in the rat femur directly activates spinal pain pathways. In their study, evoked potentials in the spinal dorsal horn were registered in 62% of wide-dynamic-range neurons and 86% of high-threshold neurons, whereas low-threshold neurons remained unresponsive [33].

The results of these studies indicate that nociceptive and sensory fibers possess a high activation threshold and are stimulated only at significant levels of hypertension. This high threshold aligns with the physiological observation that intraosseous pressure can transiently rise during movement and physical exertion under normal, healthy conditions [59]. The pathological impact of intraosseous hypertension is mediated by the activation of peripheral intraosseous nociceptors, driven either by the release of inflammatory mediators or via direct mechanical compression and distortion [60]. Moreover, this hypertension can amplify episodic pressure responses, with the subsequent intravasation of epiphyseal fat leading to bone marrow edema and altered joint mechanics [61].

Among the neural populations, Aδ intraosseous nociceptors are responsible for sensing both the intensity and the rate of pressure changes. Experimentally, an increase in intraosseous pressure induced by injecting heparinized physiological saline (0.9% NaCl) into the rat tibia evokes a differentiated pattern of activation: phasic–tonic units code for absolute intraosseous pressure levels, whereas phasic units are activated to register the rate of pressure change [62].

#### 3.1.5. Central Sensitization and Trigger Point Formation

##### Intraosseous Nociceptive Hyperexcitability

Prolonged and elevated irritation of intraosseous nociceptors, driven by degenerative and molecular alterations within the spinal bone tissue (such as endplate microfractures, localized ischemia, and pro-inflammatory cytokine upregulation), not only intensifies nociceptive input but also enhances receptor excitability to subthreshold stimuli [62,63].

**Figure 2:**
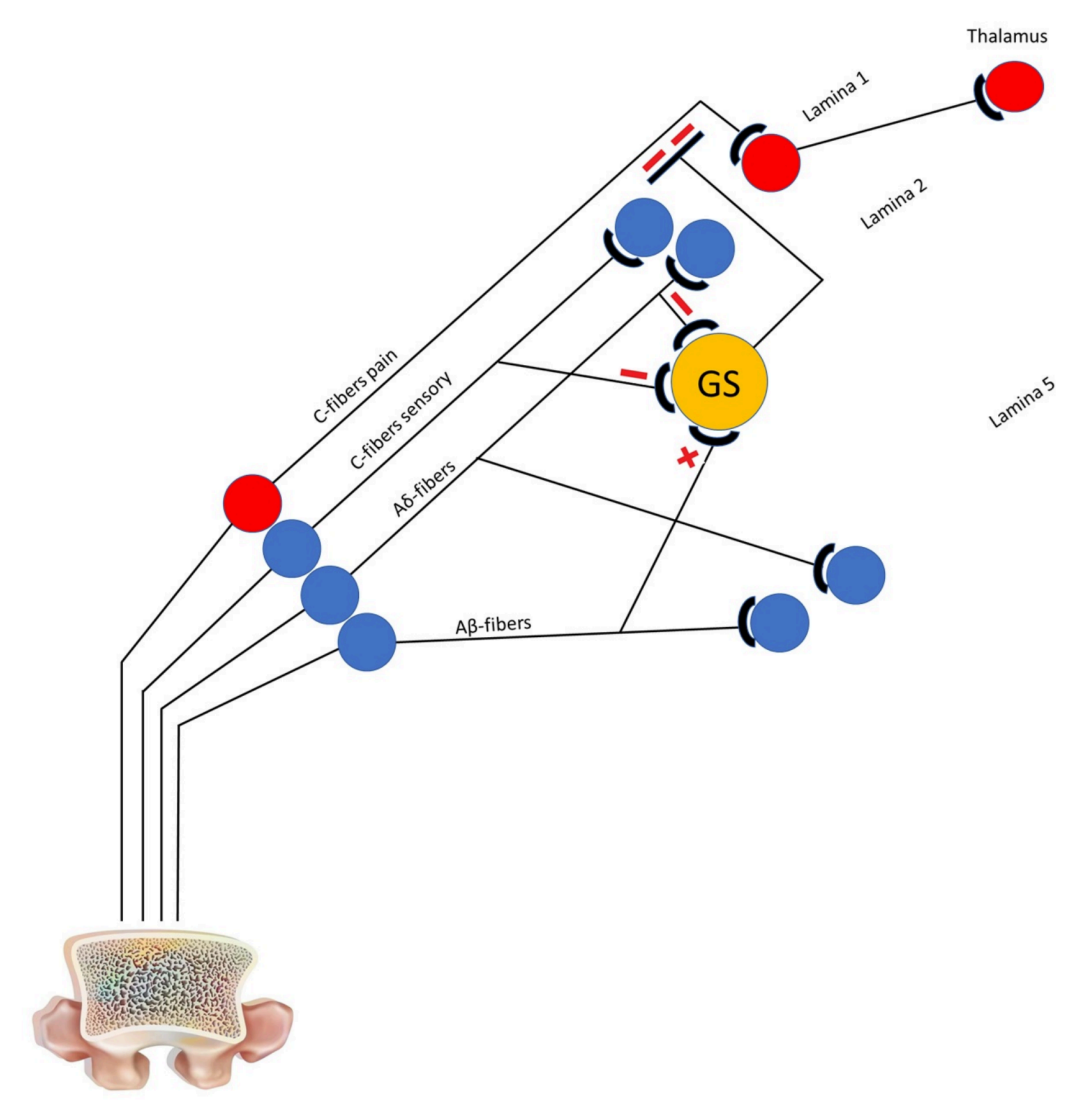
Interaction of Intraosseous Afferents within the Substantia Gelatinosa and Its Modulation of Nociceptive Inflow.

According to the gate control theory of pain, slow unmyelinated C-fibers and thinly myelinated Aδ-fibers attenuate the tonic suppressive effect of inhibitory interneurons within Lamina II (substantia gelatinosa) of the spinal cord dorsal horn [64]. Consequently, the intensified, chronic afferent influx traveling along slow non-nociceptive intraosseous afferents undergoes a functional shift. Under pathological conditions, this heightened sensory input from small non-nociceptive fibers significantly reduces the synaptic resistance of the substantia gelatinosa (gatekeeping interneurons) to intraosseous nociceptive signaling. This opens the segmental spinal cord pain gate, resulting in promoted and amplified nociceptive transmission to the brain and directly driving the mechanisms of segmental spinal and central sensitization [64,65] (Figure 2).

##### Segmental and Central Sensitization

Beyond the role of nociceptive hyperafferentation in the development of segmental spinal and central sensitization, numerous authors have identified histological and biochemical alterations within the dorsal root ganglion (DRG). Within these structures, the upregulation of several pro-inflammatory cytokines and mediators, including IL-1β, TNF-α, IL-6, and CGRP, has been observed [65,66,67]. Furthermore, the internalization and retrograde transport of NGF/TrkA receptors have been demonstrated following their binding on peptidergic nociceptors, a process which involves the upregulated expression of several NGF signaling molecules, including VGSCs and TRPV1 in the DRG [68]. Consistently, Williams demonstrated that the injection of physiological saline under pressure into the bone marrow of rats leads to activity-dependent changes in Fos protein expression within the superficial dorsal horn, thereby reflecting the processing of nociceptive input [69].

Other authors associate hyperalgesia and paresthesia within the dermatomes of the innervation zones of affected bones with the mechanisms of peripheral and central sensitization [33,70]. Hypoesthesia within the pain zone—which in many cases is misinterpreted as being of neural or radicular origin—disappears immediately following a potent analgesic intervention. This phenomenon can be explained by nondermatomal hypoesthesia resulting from the segmental inhibition of sensory afferentation due to segmental spinal sensitization [71].

##### Myofascial Trigger-Point Biology

Myofascial trigger points are palpable, hyperirritable areas within skeletal muscle that, upon palpation, provoke distant referred pain and protective muscle contractions, reproducing the patient’s activity-related pain [15,72,73]. These hyperirritable spots are characterized by a hypersensitive, palpable nodule situated within a taut band. They develop due to localized histological and biochemical alterations in reflexively active points [72,73,74]. These tissue modifications exhibit sympathetic hyperinnervation and nociceptor hyperexcitability [73,74,75]. Upon stimulation, the activity of central sensitization mechanisms is amplified, whereas their inhibition actively contributes to their suppression [76,77].

There is a close link between intraosseous nociceptor irritation and the formation of segmental protective muscular hypertonicity and myofascial trigger points through convergent spinal reflex arcs. This chronic, microvascular-restricting muscle spasm creates a localized ‘energy crisis’ characterized by hypoxia and persistent acetylcholine release, ultimately consolidating into palpable, hypersensitive myofascial trigger points within the corresponding dermatomal and myotomal distribution [78].

##### The Proposed Concept of Osseous Trigger Points

While the definition of myofascial trigger points was first introduced by authors Janet Travell in 1942 [76] and Arthur Steindler in 1940 [79], the term “osseous trigger points” was proposed only in 2024 [15]. This concept refers to findings that deep palpation of the spinous processes or percussion of certain bone tissues may induce a pain reaction similar to the reaction experienced during the palpation of myofascial trigger points. Research suggests that these localized points of bone sensitivity may play a role in the formation and amplification of neurophysiological mechanisms of central sensitization, thereby affecting the severity and distribution of the clinical manifestation of pain [80]. It is noted that clinicians utilizing intraosseous LTPI have historically used palpation of these trigger points as a diagnostic marker [15].

From a clinical perspective, the proposed concept of an ‘osseous trigger point’ functions as a highly specific diagnostic phenomenon that closely mirrors the semiology of classical myofascial trigger points. During deep digital palpation or mechanical percussion of specific osseous structures—such as the spinous processes or the PSIS—the patient exhibits a profound nociceptive response accompanied by an immediate local or segmental muscle twitch and hypertonicity. Crucially, this mechanical stimulus of these trigger points replicates the exact, recognizable pain pattern experienced by the patient during severe clinical exacerbations, often inducing secondary, segmentally linked muscular reactions. In clinical practice, these tender osseous landmarks serve as essential navigational markers for specialists executing intraosseous LTPI, as targeting these precise sites of maximum tenderness directly contributes to achieving optimal analgesic efficacy. From a pathophysiological perspective, we hypothesize that the underlying mechanism driving the formation of osseous trigger points is a state of localized hyperexcitability within the periosteal nociceptors. This phenomenon is directly linked to development secondary to sustained, high-frequency afferent bombardment from sensitized intraosseous receptors resulting from intraosseous hypertension, tissue acidosis, and underlying intraosseous degenerative and molecular alterations. This persistent up-regulation of adjacent periosteal unmyelinated fibers lowers their mechanical threshold and establishes a pathological bone-to-muscle reflex loop that manifests clinically as a somatic, recognizable pain experience in response to mechanical irritation.

### 3.2. Technique and Therapeutic Pathways of Intraosseous LTPI

#### 3.2.1. Technique of Intraosseous LTPI

**Figure 3:**
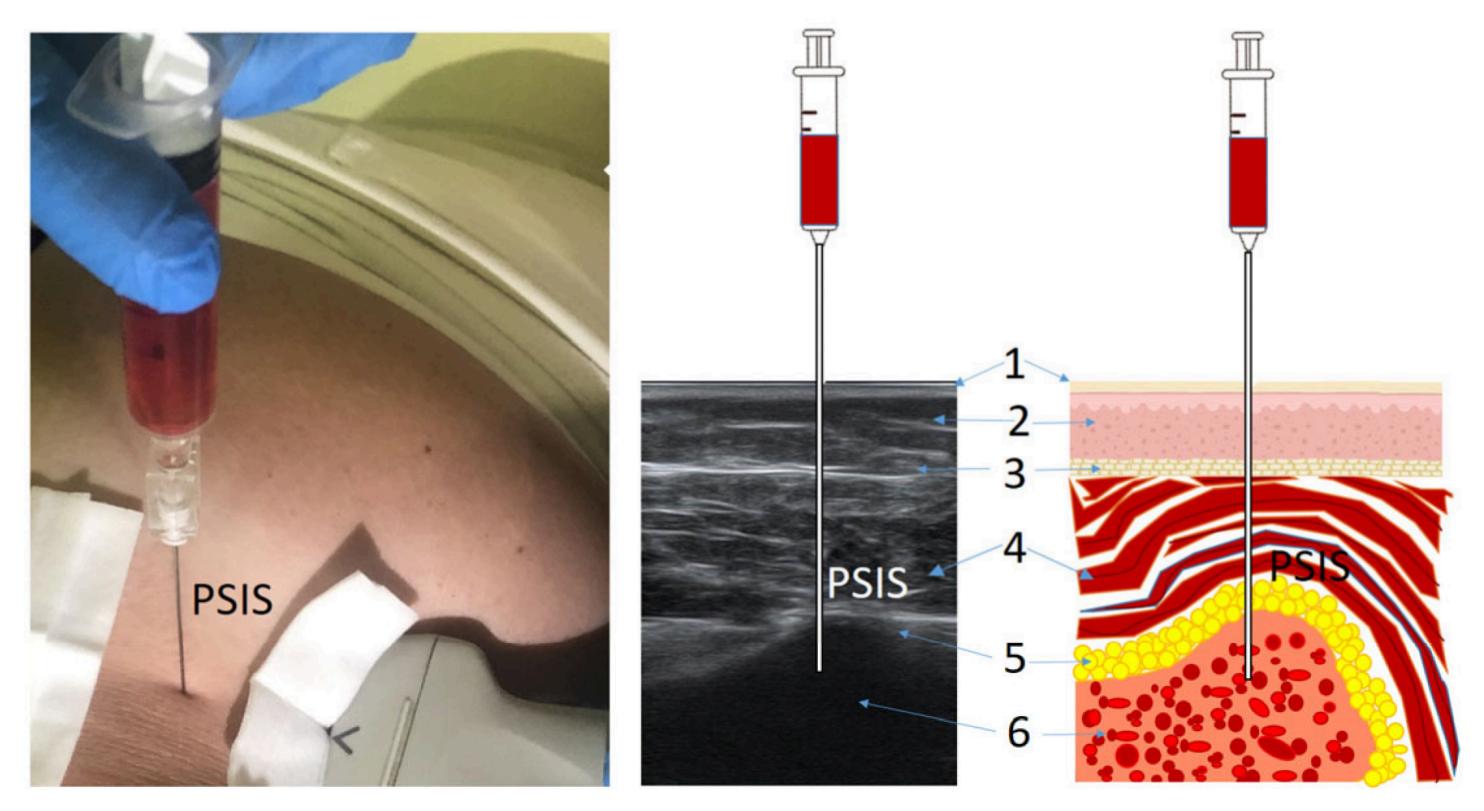
Ultrasound-guided intraosseous lidocaine trigger point injection technique. Notes: PSIS: posterior superior iliac spine; 1: epidermis/dermis; 2: subcutaneous tissue; 3: superior muscle fascia; 4: gluteus maximus; 5: periosteum; 6: spongey substance [15].

Intraosseous infusion involves the direct insertion of a needle into the cancellous bone, enabling the rapid entry of fluids and medications into the bone marrow vasculature and subsequent systemic circulation [81]. In a study of healthy adult volunteers, researchers injected contrast medium through the proximal humerus and captured with fluoroscopy as it entered the heart. The mean time to reach the contrast in the superior vena cava and the right atrium was 2.42 s [82]. To ensure methodological transparency, safety, and reproducibility, the procedure must be executed under strict aseptic and antiseptic conditions utilizing a stylet-guided (trocar-tipped) needle. Needle insertion is primarily guided by the palpation of prominent bony landmarks. However, in challenging clinical scenarios—such as patients presenting with obesity or severe anatomical deformities—ultrasound-guided control can be utilized to ensure precise targeting [15] (Figure 3).

#### 3.2.2. Step-by-Step Puncture and Injection Protocol

Due to the invasive nature of this technique, it is strongly recommended that medical specialists are not permitted to perform this procedure without specialized, structured training. To address this requirement, dedicated postgraduate training curricula have been developed. For instance, at RUDN University, this specialized postgraduate training program comprises 72 academic hours of theoretical and practical instruction, ensuring adequate clinical competency before independent practice.

The standard clinical protocol for intraosseous LTPI comprises the following sequential steps [15,83,84,85,86]:

- Identification of the Osseous Trigger Points: Deep palpation of the spinous processes is performed within the painful region to identify the most tender bone trigger point that elicits a characteristic local twitch response. Importantly, while these trigger points are identified superficially on the bone and periosteum, they serve as crucial anatomical markers for the subsequent intraosseous injection. Additionally, the tenderness of adjacent accessory trigger zones is evaluated—specifically focusing on the scapular spine regions in cases of cervicalgia, and the PSIS in patients presenting with low back pain.
- Aseptic Preparation and Navigation Guidance: The procedure must be performed under strict aseptic and antiseptic conditions utilizing a specialized stylet-guided (trocar-tipped) intraosseous needle. While needle trajectory and insertion are primarily guided by the manual palpation of the previously identified bony landmarks, advanced imaging modalities (ultrasound-guided control navigation) can be integrated to enhance accuracy (Figure 3) [15].
- Cortical Penetration: The needle, containing its stylet, is advanced perpendicularly through the skin and subcutaneous tissue until it contacts the periosteum, which typically elicits a mild, localized pain sensation. Following this, using a rotational, advancing motion, the needle penetrates the dense cortical bone to reach the cancellous bone tissue.
- Verification of Needle Placement: Entry into the spongy bone marrow space is signaled by a distinct “give” or a sudden drop in resistance. The stylet is then removed, and a syringe containing the medication is attached.
- The Aspiration Test: To confirm correct placement within the cancellous bone, an aspiration test is performed. A successful test must yield a free flow of bone marrow into the syringe without resistance.
- Preparation and Injection of the Therapeutic Agent: The contents of the syringe are mixed with 2 mL of blood and 4 mL—2% [15], 2%—2 mL [86] or 8 mL—1% lidocaine solution [83,84,85] with 1–2 mg dexamethasone or without, and then slowly injected into the cancellous bone. During this injection, patients frequently experience a transient pain or pressure sensation that mimics the characteristic exacerbation of their baseline pain syndrome. This phenomenon serves as an additional clinical indicator of correct needle positioning.
- Post-procedural Care: Upon completion of the injection, the needle is carefully withdrawn. The puncture site is immediately treated with an antiseptic solution, and a sterile occlusive dressing is applied.

##### Technical Challenges, Success Rates, and Procedural Complications of Intraosseous LTPI

The success rate for intraosseous access—often defined by the successful penetration of the cortical bone—is generally reported to be high, ranging from 71% to 100% in various clinical studies [87,88]. High success rates are typically associated with experienced clinicians and proper training. Furthermore, clinical guidelines suggest that the number of insertion attempts should be limited to avoid unnecessary trauma; the literature indicates that successful bone penetration typically occurs on the first attempt, with a maximum of up to three puncture attempts [88]. Notably, studies demonstrate the success rate of intraosseous access exceeding 90%, with infusion initiation typically occurring within 60 s, which is significantly faster than peripheral or central venous catheterization [89]. However, according to Hallas, difficulties with penetration of the periosteum were reported in 10.3% of cases, while difficulties with bone marrow aspiration occurred in 12.3% of cases. Additionally, technical complications included difficulties with the injection of fluids and drugs after insertion in 7.4% of cases, slow infusion rates in 8.8%, and needle displacement after insertion in 8.5% of cases [88].

The majority of published studies report no serious adverse effects associated with intraosseous injections. The most common complication is procedure-related pain, the incidence of which varies across the literature. While some authors recorded procedural pain in only 7.1% of cases [88], others reported a nearly tenfold higher incidence, with moderate-to-severe pain affecting approximately 65% of patients during cortical penetration and 73% during the flushing phase [89].

Several authors consider the pain experienced during intraosseous injection to be a physiological response rather than a pathological complication. Previous research confirms that the dominant pain source during cortical penetration is attributed to the richly innervated, highly sensitive periosteum, whereas the traction-type pain experienced during fluid flushing is driven by an abrupt elevation of intraosseous pressure [89]. While many investigators registered no major complications during intraosseous interventions, critical adverse events have been documented in larger cohorts. Specifically, an analysis of 1,802 clinical cases revealed that compartment syndrome occurred in 0.6% of cases, while osteomyelitis was diagnosed in 0.4% of cases [88].

To mitigate the risk of such severe adverse events and prevent complications associated with intraosseous trigger point injections, a rigorous pre-procedural screening is mandatory. The intervention is strictly contraindicated in patients presenting with the following conditions [83,84]:

- Febrile conditions of infectious origin and acute respiratory infections (ARIs).
- Hematological and coagulation disorders.
- Oncological conditions and malignant neoplasms.
- Systemic and local infections.
- Decompensated organ failure.
- Allergic hypersensitivity to any component of the injectate.
- Acute gastrointestinal inflammatory diseases.

#### 3.2.3. Therapeutic Pathways of Intraosseous LTPI

##### Pharmacological Mechanisms and Molecular Targets of Lidocaine Administration

Although direct evidence regarding the molecular mechanisms of lidocaine specifically during intraosseous administration remains sparse in the current literature, extensive experimental research has uncovered a comprehensive multi-target pharmacological profile within other tissues. This complex mechanism can be characterized by three primary distinct therapeutic pathways: a potent analgesic effect, a robust anti-inflammatory effect, and a specialized sympatholytic-like effect.

First of all, lidocaine acts as a molecular chaperone that rescues NaV1.8 channels from endoplasmic reticulum retention, significantly increasing their functional cell surface expression. Lidocaine-induced channels exhibit biophysical properties comparable, though not identical, to native tetrodotoxin-resistant (TTX-R) DRG currents. Lidocaine thereby drives this upregulation by directly facilitating efficient intracellular forward trafficking and functional membrane integration of NaV1.8 [90]. Furthermore, following lidocaine treatment in a rat model of spared nerve injury, a significant reduction was observed in the aberrant expression of both NaV1.7 and NaV1.8 channels induced by peripheral nerve injury [91]. Lidocaine stabilizes hyperexcited neural membranes by blocking voltage-insensitive “flicker” K+ and inward rectifier Kir2.x channels, while suppressing pathological neurotransmitter release via voltage-gated calcium channel (VGCC) inhibition [92,93].

One of the primary mechanisms of lidocaine-induced local anesthesia involves the reversible inhibition of action potential propagation via the blockade of voltage-gated sodium channels (VGSCs). Structurally, only the un-ionized (free base) lipophilic form of the drug possesses the capability to permeate the neuronal lipid bilayer. Upon entering the intracellular compartment, the re-ionized form fractionally binds to the specific receptor site located at Segment 6 of Domain IV (S6/DIV) within the pore-forming alpha-subunit of the channel. This binding event physically obstructs the transmembrane influx of Na^+^ ions, thereby suppressing subsequent depolarization, action potential generation, and saltatory conduction. Notably, lidocaine exhibits state-dependent binding kinetics, displaying a preferential affinity for the open or inactivated conformations of VGSCs. Consequently, the onset of its inhibitory effect is pronouncedly accelerated in rapidly firing, highly active nociceptive fibers [93]. Crucially, lidocaine modulates and inhibits NMDA receptors, directly interfering with the transmission of persistent pain signals and thereby dampening the central sensitization cascades [93].

Lidocaine exerts a profound anti-inflammatory effect by interrupting molecular cascades and neurogenic inflammation. Experimentally, at nanomolar concentrations, lidocaine acts as a potent intracellular inhibitor of Gαq protein signaling via m1 and m3 muscarinic receptors, interrupting downstream pro-inflammatory pathways [94]. Additionally, local anesthetics possess neurotrophin receptor kinase A (TrkA) inhibitory effects, and lidocaine has been shown to directly suppress the mRNA expression and protein levels of nuclear factor kappa B (NF-κB) and tumor necrosis factor-alpha (TNF-α) [92]. This action effectively inhibits the active inflammatory process during musculoskeletal injuries. This anti-inflammatory profile underscores a critical pathophysiological synergy: the direct electrophysiological blockade of sodium channels works in tandem with the mitigation of inflammatory TRPV1 channel sensitization [95].

**Figure 4:**
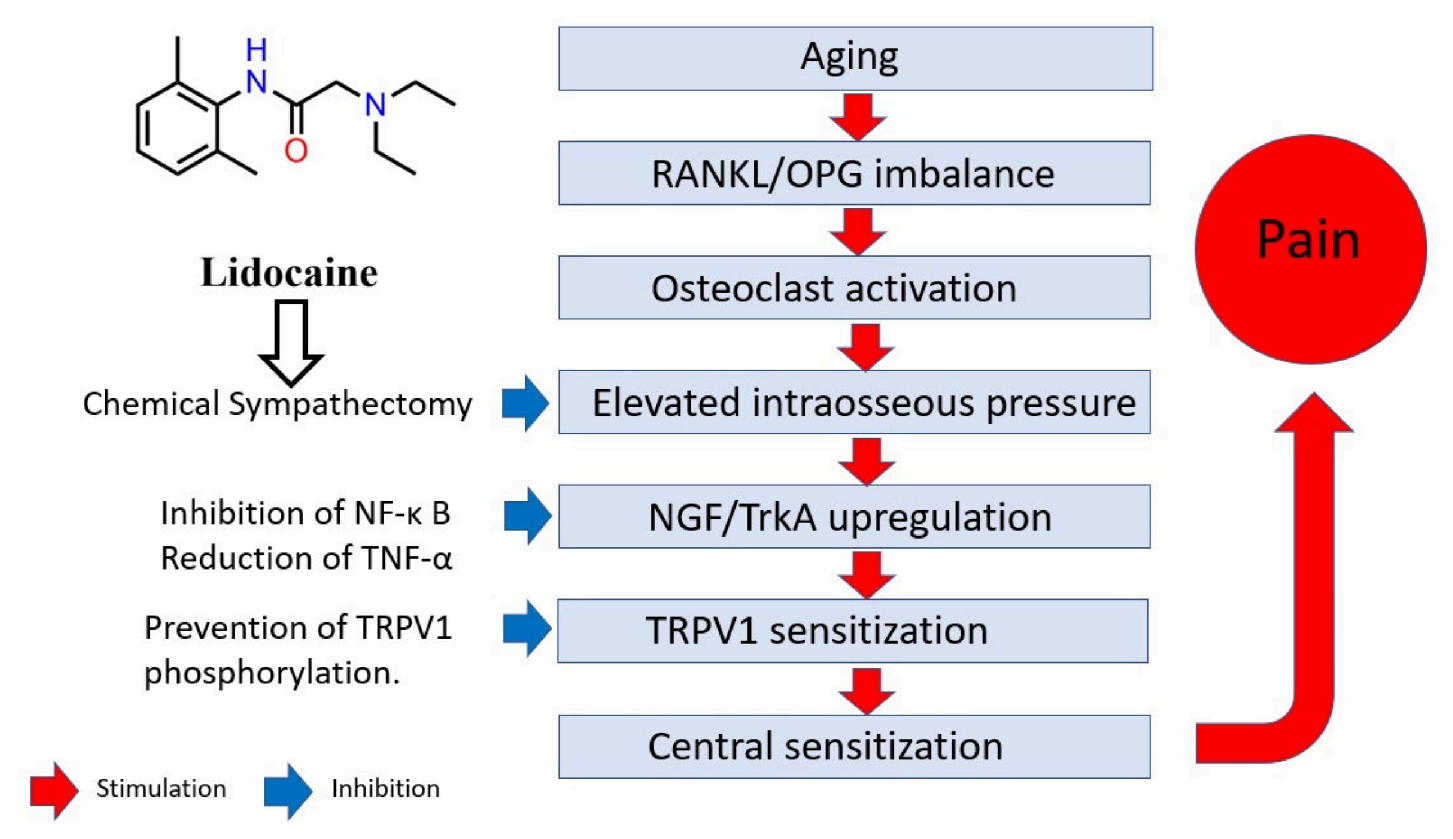
Integrative mechanistic cascade of aging-related vertebral degeneration and the multi-node mechanism of intraosseous lidocaine action. Notes: NF-kappa B—Nuclear factor kappa-B, NGF—Nerve growth factor, OPG—Osteoprotegerin, RANKL—Receptor activator of nuclear factor-kappa B ligand, TNF-α—Tumor necrosis factor-alpha, TrkA—Tropomyosin receptor kinase A, TRPV1—Transient receptor potential vanilloid 1.

Furthermore, lidocaine demonstrates a distinct sympatholytic-like effect that targets neuroplastic and vascular complications of chronic pain. Evidence indicates that sympathetic sprouting near sensory neurons after nerve injury develops preferentially around spontaneously active cells, a phenomenon that is effectively mitigated by lidocaine blockade [91]. Although this mechanism remains hypothetical in the context of the intraosseous microenvironment, a similar cascade may occur during the action of lidocaine on intraosseous sympathetic receptors. Through silencing of the spontaneously active bone pain pacemaker, early lidocaine blockade may suppress this aberrant sympathetic sprouting, potentially contributing to local venodilation and the subsequent reduction in intraosseous hypertension (Figure 4).

##### Pharmacological Mechanisms and Molecular Targets of Dexamethasone Administration

Beyond its classic anti-inflammatory activity, dexamethasone downregulates pro-inflammatory cytokines, such as interleukin-8 (IL-8), by driving targeted shifts in cellular bioenergetics [96]. Specifically, dexamethasone reduces glucose transporter GLUT3 expression and enhances PKM2 levels through hydrogen bonding with fructose-1,6-bisphosphate (FBP), while concurrently downregulating the mitochondrial outer-membrane protein mitofusin-2 (Mfn2) [96]. Furthermore, dexamethasone delivery provides extensive microenvironmental stabilization by inhibiting matrix metalloproteinases (MMP2, MMP13), fibrotic markers (COL3A1, bFGF), and osteogenesis-related genes (RUNX2, BMP2, TNFRSF11B) involved in pathological tissue remodeling [97]. Collectively, these highly localized molecular cascades reverse the hyperexcitability of TrkA, p75, and ASIC3 bone nociceptors, resolve trabecular micro-edema, and lower intraosseous pressure.

According to the established systemic and perioperative literature, weight-based low-dose intravenous dexamethasone at a threshold of 0.1 mg/kg is recognized as the clinical standard required to achieve effective anti-inflammatory and multimodal analgesic outcomes [98]. However, when it comes to the intraosseous administration of this agent, no empirical data regarding local pharmacokinetics, tissue concentration, or anti-inflammatory thresholds could be identified or recorded in the literature.

Nevertheless, evidence in clinical dental anesthesia indicates that even at these localized concentrations (2 mg), dexamethasone synergistically enhances the local anesthetic action of 2% lidocaine [99].

##### Bone Cortical Decompression

Mechanical bone cortical decompression in intraosseous LTPI is hypothesized to be driven by the physical creation of drainage pathways within the cortical layer of the targeted bone structures. During the execution of intraosseous puncture, specialized trocars with a stylet (mandrin) are utilized. As a result, localized channels are formed directly leading into the cancellous bone cavity, with an individual diameter depending on the size of the needle [15,83,84,85,86]. Furthermore, it cannot be excluded that performing a sequential course of several treatment procedures may develop an effective multi-channel network with small diameters. Hypothetically, the total surface area of these small-diameter channels functions as a cumulative decompression tract, establishing a potential decompression approach for pathological intraosseous hypertension and venous engorgement. It is suggested that this specific technique effectively mimics the biomechanical principles of multiple small-diameter drilling decompression (multiple drilling), a well-established joint-preserving surgical modality actively utilized in advanced clinical practice for the direct management of avascular bone necrosis (osteonecrosis) and severe, treatment-resistant intraosseous pressure elevation [100]. Crucially, the subsequent reduction in intraosseous pressure is hypothesized to exert a dual therapeutic impact.

On one hand, it may potentially optimize the bone reparative function and mitigate localized osteoporosis by restoring capillary perfusion, reducing micro-ischemia, and balancing the RANKL level to suppress pathological osteoclastogenesis [41].

On the other hand, it is suggested that by lowering intraosseous pressure, decompression achieves the deactivation of fine-diameter afferent nociceptors in the bone marrow and suppresses downstream Fos expression within superficial spinal dorsal horn neurons. At the molecular level, this pressure reduction neutralizes the mechanical noxious stimuli that synergize with inflammatory chemical mediators—specifically hydrogen (H^+^) ions, potassium (K^+^) ions, histamine, and bradykinin—thereby blocking the ionic and receptor-mediated signaling pathways that excite these nociceptive fibers. By preventing the threshold activation of these intraosseous nerve endings, decompression halts the local and systemic nociceptive cascades and clinically alleviates both acute and chronic musculoskeletal pain [33].

##### Hypothetical Role of Autologous Blood Injection

Autologous Blood Injection (ABI) is a localized injection therapy that involves introducing a patient’s own venous blood directly into inflammatory foci. This technique aims to stimulate a localized immune response and induce regenerative processes, ultimately facilitating the reduction in the inflammatory process in the target zone [101].

A critical technical parameter of intraosseous LTPI shared across all reviewed clinical protocols is the immediate, pre-injection mixture of the local anesthetic with a small volume (approximately 1–2 mL) of bone marrow aspirated from the cancellous bone for subsequent reinjection into the same cancellous bone [15,83,84,85,86]. From a technical standpoint, this autologous component is not an isolated, optional pharmacological add-on, but rather an inherent and inevitable consequence of the intraosseous access procedure itself. The clinical gold standard for verifying correct needle placement within the vertebral or pelvic cancellous bone is a positive aspiration test. The physical act of aspiration causes the immediate entry of autologous blood and cancellous bone marrow contents into the syringe, which dynamically mix with the lidocaine solution prior to final fluid delivery.

In this context, a clear distinction must be made between this procedural phenomenon and conventional autologous blood therapies or platelet-rich plasma (PRP) therapy, which fundamentally differ in both technical execution and primary therapeutic intent. During intraosseous LTPI, the aspirate consists of unmodified whole blood and bone marrow elements drawn directly from the cancellous bone and reinjected within seconds. Therefore, this specific technique cannot be conceptually classified as a specialized form of autologous blood injection or an independent biological treatment. Because this aspirated volume is immediately returned to its native microenvironment, its transient mixture with lidocaine and dexamethasone serves primarily as a procedural safety indicator to confirm correct intraosseous needle positioning, rather than a standalone therapeutic intervention.

### 3.3. Clinical Applications of Intraosseous Lidocaine Trigger Point Injection in the Treatment of Vertebrogenic Pain

The analysis of the scientific literature over the last 20 years reveals a notable scarcity of global publications. This phenomenon can be primarily attributed to limited accessibility, as the vast majority of pioneering and foundational studies have been published in non-open-access journals and in the Russian language.

#### 3.3.1. Low Back Pain

The application of intraosseous LTPI for the treatment of low back pain has been described in several scientific works [30,102,103]. One of the studies conducted in 2023 was a pilot study applying intraosseous LTPI in the treatment of 13 patients with severe LBP [86]. The procedure involved injecting a mixture of 2 mL of 2% lidocaine and 2 mg of dexamethasone into the most painful trigger points within the lumbosacral region, which were localized in the projection of the L5 and S1 spinous processes, as well as the posterior superior iliac spine (PSIS). Following the treatment course, patients demonstrated significant improvements, including a 44.3% (*p* < 0.05) reduction in Visual Analogue Scale (VAS) pain scores and a 40.2% (*p* < 0.05) improvement in disability index. Long-term monitoring revealed exceptional therapeutic stability in the intraosseous LTPI group, with 100.0% of patients exhibiting no negative clinical dynamics at the 2-month follow-up, and 53.8% (*p* < 0.05) of patients maintaining stable, positive outcomes at the 3–6-month follow-up. Neither technical impediments during the procedure nor any adverse sequelae were reported following intraosseous LTPI administration. It is methodologically important to note that the primary clinical study by Kornilova et al., 2023 [86] utilized a comparative multi-arm framework designed to evaluate the clinical efficacy of combining intraosseous blockades with intramuscular injections of the HYALREPAIR-02 bio-reparant formulation (hyaluronic acid modified with vitamin C and amino acids) against a standard intraosseous therapy baseline. The active control group in their trial received isolated intraosseous LTPI containing a mixture of 2%—2 mL lidocaine and 2 mg dexamethasone as a monotherapy. Because the strict eligibility scope of the present narrative review is focused exclusively on the therapeutic impact and physiological mechanisms of lidocaine-based intraosseous trigger point injections, we intentionally extracted and analyzed quantitative clinical data solely from this active lidocaine/dexamethasone control cohort (n = 13), while omitting the parallel chondroprotector combination arm to preserve synthesis specificity.

#### 3.3.2. Vertebrogenic Cervicobrachialgia

In 2011, a comparative study was conducted between intraosseous LTPI and intramuscular LTPI for the treatment of patients with vertebrogenic cervicobrachialgia. In this study, 70 patients underwent intraosseous LTPI, while 40 patients received paravertebral intramuscular LTPI [85]. The intraosseous LTPI protocol consisted of five procedures administered every other day, injecting a mixture of 8 mL of 1% lidocaine and 1 mg of dexamethasone targeted into the spinous processes of the C2, C6, C7, and T1 vertebrae, as well as the scapular spines. In the control group, paravertebral intramuscular LTPI was administered into the myofascial trigger points paravertebrally at the corresponding anatomical levels. Post-treatment analysis demonstrated the clinical superiority of intraosseous LTPI over the control group, yielding a 43.1% (*p* < 0.05) greater reduction in VAS pain scores, as well as a 27.1% (*p* < 0.05) and 9.7% (*p* < 0.05) greater decrease in the sensory and affective dimensions of the McGill Pain Questionnaire (MPQ) Pain Rating Index (PRI), respectively. Furthermore, intraosseous LTPI achieved a 66.1% greater improvement in shoulder range of motion (ROM) restriction compared to paravertebral intramuscular LTPI. Long-term clinical outcomes were not assessed. Intraosseous LTPIs were executed without any technical challenges or subsequent clinical complications.

Sokov E.L. et al. (2014) conducted a single-group pre-test/post-test study investigating the efficacy of intraosseous LTPI in 50 patients suffering from neck pain [83]. The therapeutic intervention involved a course of five alternating-day procedures, during which 8 mL of 1% lidocaine and 1 mg of dexamethasone were injected into the spinous processes of C2, C6, C7, T1, and the scapular spines. Following the treatment course, significant therapeutic benefits were observed. VAS pain scores dropped by 59.7% (*p* < 0.05), while the sensory and affective dimensions of the MPQ decreased by 48.1% (*p* < 0.05) and 70.0% (*p* < 0.05), respectively. Long-term follow-up outcomes were not monitored in this study. No peri-procedural difficulties or untoward effects were observed during or after intraosseous LTPI.

#### 3.3.3. Residual Chronic Pain After Degenerative Lumbar Spinal Stenosis Decompression Surgery

The largest study evaluating intraosseous LTPI was a 2024 randomized controlled trial (RCT) by Al-Zamil M. et al., which investigated therapeutic strategies for residual chronic pain after degenerative lumbar spinal stenosis decompression surgery [15]. The study cohort comprised five arms: intramuscular LTPI at L4–S1 (n = 22), intramuscular LTPI in the PSIS region (n = 22), intraosseous LTPI at the L5 or S1 spinous processes (n = 22), intraosseous LTPI into the PSIS (n = 23), and a control group receiving standard pharmacotherapy (n = 21). The LTPI protocol involved five procedures at 3-day intervals, injecting 4 mL of 2% lidocaine into the respective osseous structures or myofascial trigger points. Direct comparison showed that intraosseous LTPI significantly outperformed intramuscular LTPI, yielding a 57.1% (*p* < 0.01) greater reduction in VAS pain, a 26.3% (*p* < 0.01) greater decrease in tactile hypoesthesia, and a 36.4% (*p* < 0.05) superior improvement in claudication disorders as determined by the Zurich Claudication Questionnaire (ZCQ). Within the intraosseous arms, the PSIS intervention showed a clear advantage over the spinous process approach, demonstrating a 50.0% (*p* < 0.01) greater reduction in VAS pain, a 37.3% (*p* < 0.01) greater decrease in tactile hypoesthesia, and a 35.1% (*p* < 0.05) superior improvement in reducing claudication disorders. The intraosseous LTPIs were well-tolerated, with no technical difficulties or adverse clinical outcomes reported. Short-term follow-up confirmed sustained relief compared to baseline. At 2 months, a 62.9% (*p* < 0.01) reduction in VAS pain, a 52.9% (*p* < 0.01) decrease in tactile hypoesthesia, and an improvement in reducing claudication disorders by 42.7% (*p* < 0.01) were maintained. At 4 months, positive dynamics persisted despite a gradual return of symptoms, showing a 38.5% (*p* < 0.01) decrease in VAS pain, a 25.9% (*p* < 0.05) reduction in tactile hypoesthesia, and an improvement in reducing claudication disorders by 30.7% (*p* < 0.05) compared to baseline.

In a single-group pre-test/post-test study by Sokov et al. (2008), 44 patients presenting with Lumbar Failed Back Surgery Syndrome underwent a course of five intraosseous LTPIs [84]. Patients received injections of 8 mL of 1% lidocaine combined with 1 mg of dexamethasone into the spinous processes of L4, L5, and S1, the posterior superior iliac spine, and the fibular head every other day. Following the treatment protocol, patients demonstrated significant clinical improvements. Visual Analog Scale (VAS) pain scores decreased by 45.7% (*p* < 0.05). Pain evaluation via the McGill Pain Questionnaire (MPQ) revealed a 43.4% (*p* < 0.05) reduction in the sensory Pain Rating Index (PRI) and a 38.9% (*p* < 0.05) decrease in the affective PRI. Additionally, the pain drawing extent contracted sharply by 87.3% (*p* < 0.05). However, the study noted that long-term clinical outcomes were not assessed. No procedural technical difficulties or adverse complications were recorded during the administration of intraosseous LTPI (Table 1).

## 4. Discussion

Despite the long-standing and widespread application of intraosseous local anesthetics, including lidocaine, in dental anesthesia [104], intraosseous LTPIs have not achieved global adoption as a therapeutic modality for chronic vertebrogenic pain. For a considerable duration, this clinical practice remained geographically isolated, localized primarily within Russia, USA, Eastern Europe, Armenia, Tajikistan, and Uzbekistan. This limited dissemination is largely attributable to a historical lack of English-language publications in high-impact journals, particularly a scarcity of robust randomized controlled trials (RCTs). Furthermore, a significant barrier to the global adoption of this method is the poorly understood role of intraosseous receptors in the pathogenesis of chronic pain syndromes. This scarcity of literature is further compounded by prevailing clinical misconceptions that intraosseous injections are inherently painful, hazardous, and associated with a high risk of severe complications. Accordingly, this narrative review sought to demonstrate and bridge the gaps in the limited and historically fragmented literature surrounding the evolution of intraosseous nociception science, with a specific focus on its role in chronic pain pathogenesis across macroscopic, microscopic, and molecular levels, as well as the clinical efficacy of intraosseous LTPI in patients with vertebrogenic pain.

### 4.1. From Structural Bone Degeneration to Molecular Nociception

**Figure 5:**
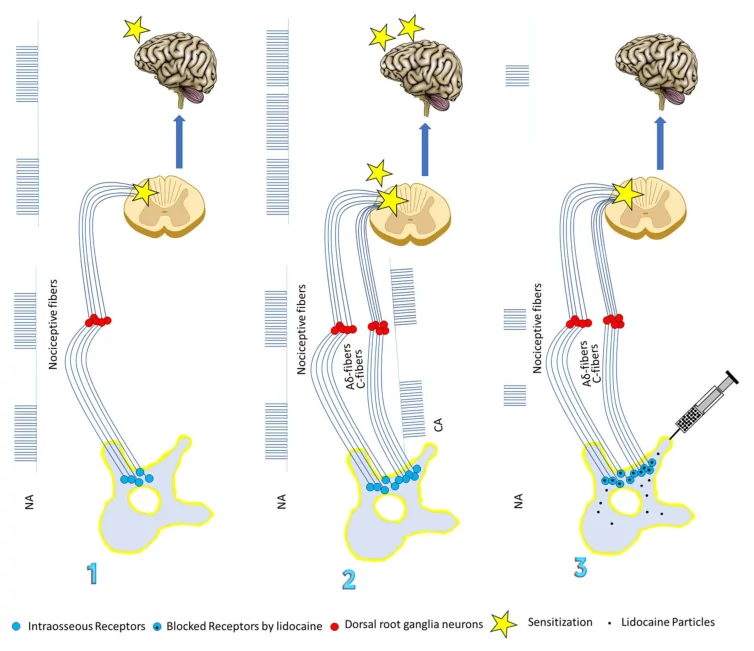
Molecular and neurophysiological mechanisms of the analgesic effect of intraosseous lidocaine administration. 1. Activation of Intraosseous Nociceptors: Pathological excitation of intraosseous nociceptors generates an excessive afferent influx into the substantia gelatinosa (Lamina II) of the spinal cord and subsequently via ascending pathways to the brain, driving the progressive development of segmental and central sensitization. 2. Pathological Modulation of Nociceptive Inflow: Chronic irritation of slow-conducting sensory intraosseous receptors enhances the propagation of impulses through the spinal dorsal horn to the brain. This occurs via the suppression of inhibitory interneurons within the substantia gelatinosa, which normally exert a tonic breaking effect on nociceptive afferentation. 3. Mechanism of Intraosseous Lidocaine Blockade: Intraosseous injection of lidocaine into trigger points induces the concurrent inhibition of both nociceptive and slow sensory afferents. This produces a dual therapeutic effect: first, through direct antinociception via the blockade of intraosseous nociceptors, and second, through an indirect segmental reduction in pain by reversing the pathological suppression of the substantia gelatinosa. Notes: NA—nociceptive afferentation, CA—Slow sensory afferentation.

For decades, bone tissue and its sensory structures remained under-researched. Only since the mid-20th century have the physiological, regenerative, and plastic regulatory mechanisms of the bone microenvironment been elucidated, explaining how intraosseous receptors react to evolving osseous pathologies [63,105]. Despite the limited volume of available high-impact publications, current evidence indicates a dual-action mechanism driven by heightened afferent signaling from irritated intraosseous nociceptors. On one hand, these hyperactive receptors serve as a direct peripheral source of localized bone pain. On the other hand, according to the gate control theory of pain, this persistent nociceptive bombardment disinhibits the dorsal horn by suppressing the inhibitory interneurons of the substantia gelatinosa, thereby facilitating segmentally mediated pain transmission along concurrent nociceptive pathways [105,106]. This neurophysiological framework provides a robust pathogenic rationale for the therapeutic efficacy of intraosseous LTPI, specifically focusing on CPVP and RLVP. It is critical to differentiate this mechanism from conventional modalities; while the analgesic effects of physical therapy, acupuncture, and localized counter-irritation are primarily achieved by enhancing non-nociceptive afferent input via fast-conducting fibers (A-beta), intraosseous LTPIs are hypothesized to represent a distinct interventional approach aimed at inhibiting the slow-conducting Aδ and C-fibers that exert a disinhibitory, pro-nociceptive influence on the substantia gelatinosa (Figure 5) [11,15].

Age-related degenerative bone alterations combined with chronic mechanical stress disrupt physiological bone remodeling and compromise the homeostatic balance between osteoclasts and osteoblasts across distinct osseous regions. This dysregulation culminates in diametrically opposed structural sequelae: localized osteophyte formation in high-stress zones and osteoporotic degradation in others [107,108]. Intraosseous hypertension, alongside progressive microstructural degeneration, triggers persistent mechanical and chemical irritation of intraosseous nociceptors [33]. Simultaneously, compensatory regenerative pathways are upregulated, mediated heavily by neurotrophic factors such as NGF; when overexpressed, these factors inadvertently act as potent hyperalgesic stimuli for intraosseous nerve endings [55]. Ultimately, this sustained, low-grade nociceptive afferent bombardment drives the hyperexcitability of corresponding dorsal root ganglia, culminating in the elicitation of both spinal segmental sensitization and generalized central sensitization [64,65].

When discussing the obtained results, it is critical to first highlight the profound scarcity of scientific literature addressing intraosseous receptors. To date, the molecular mechanisms governing the interaction between inflammatory mediators and the intraosseous receptors, as well as the subsequent intracellular biochemical alterations in response to various inflammatory agents, remain virtually unexplored. Furthermore, the functional integration of periosteal and intraosseous receptors has been largely overlooked. In several fundamental areas, there is a total lack of compelling evidence supported by histological or biochemical analyses. A striking example of this data deficit is the pathogenesis of impaired venous outflow from bone tissue resulting from bone pathology and the subsequent narrowing of the efferent (emissary) channels, which serves as the primary driver of intraosseous hypertension.

### 4.2. Methodological and Molecular Gaps of Intraosseous LTPI

Given the existing clinical experience with this treatment modality, the corresponding publication activity remains remarkably scarce. Available data are predominantly localized within the regional biomedical literature, with a substantial body of these publications featuring significant historical latency. Nevertheless, and despite the absence of international consensus conferences or standardized guidelines dedicated to this method, the technical execution of intraosseous LTPI remains highly consistent across various authors.

However, a critical limitation in the existing literature is that procedural techniques are frequently described only in brief paragraphs, completely lacking granular tactical details. More importantly, the underlying mechanisms of action of intraosseous LTPI remain virtually unexamined to date. Even the specific pharmacodynamic and molecular effects of combining lidocaine and dexamethasone at the intraosseous level are significantly under-researched. Preliminary data indicate that the adverse event profile for this intervention appears to be minimal, consistent with its potential safety and clinical viability. However, the number of these studies remains scarce, often characterized by insufficient sample sizes to confidently establish the safety profile and potential adverse effects of this method. To achieve this, further research in this direction is warranted, utilizing large-scale, multicenter randomized clinical trials.

### 4.3. Evaluation of Clinical Trials Investigating Intraosseous LTPI for Vertebrogenic Pain

#### 4.3.1. Clinical Synthesis of Published Studies Across Vertebrogenic Syndromes

The clinical evidence synthesized in this review highlights the promising therapeutic feasibility of intraosseous LTPI in the management of various vertebrogenic pain syndromes.

An evaluation of the reviewed literature reveals that the use of intraosseous LTPI for low back pain (LBP) has been recently explored by Kornilova (2023) [86]. This study suffers from methodological limitations, including a small sample size and a remarkably short follow-up period. Furthermore, this work did not include a concurrent control group without intraosseous LTPI application. Importantly, more extensive investigations featuring larger patient cohorts and extended follow-up periods were conducted much earlier within the foundational PhD theses of E.L. Sokov (1985) [30] and M. Al-Zamil (2004) [103]. Both authors successfully demonstrated the clinical efficacy of intraosseous LTPI in the treatment of patients with LBP, establishing a strong historical precedent for the method.

In a study on vertebrogenic cervicobrachialgia conducted by E.L. Sokov in 2011 [85], certain limitations were found, including non-comparable sample sizes and short-term follow-up. Although their findings demonstrated the efficacy of intraosseous LTPI when administered into the spine of the scapula and the C5–C7 cervical spinous processes, a direct comparative analysis between these target sites has not been performed. Furthermore, in a subsequent study conducted by the author in 2014, the efficacy of intraosseous LTPI was evaluated in patients suffering from cervicalgia without pain radiation into the upper extremity. However, this investigation presents certain limitations, such as an uncontrolled design and a complete absence of follow-up observation [83].

Furthermore, a critical gap exists in the literature addressing residual chronic pain after degenerative lumbar spinal stenosis decompression surgery. In previously published work from 2011, data are limited by a lack of precise MRI data regarding the severity of stenosis in patients with failed decompression surgery, and the absence of a comparative analysis between treatment outcomes in patients with failed versus successful decompression surgery [84].

In contrast, the randomized controlled trial conducted by Al-Zamil et al. (2024) exclusively enrolled patients who underwent successful degenerative lumbar spinal stenosis decompression surgery—specifically, individuals without recurrent stenosis or persistent spinal canal narrowing post-operatively [15]. To confirm successful decompression and rule out residual pathology, lumbar spine magnetic resonance imaging and lower extremity electromyography studies were utilized. Through a sophisticated multi-arm comparative framework, the authors differentiated the clinical efficacy of targeting the PSIS versus the lumbar spinous processes (L4, L5, S1). Their findings demonstrated the superiority of the PSIS approach, showing significant improvements not only in terms of the analgesic effect but also in the recovery of sensory functions and the regression of neurogenic claudication. Nevertheless, the main limitations of this study remain its short-term follow-up and relatively small sample size.

#### 4.3.2. Critical Methodological Appraisal of the Evidence Base

To accurately interpret the reported clinical efficacy and safety of intraosseous LTPI, a study-level methodological appraisal of the five included publications is warranted. Structurally, the compiled evidence base exhibits substantial heterogeneity in study designs and follow-up protocols. Only one trial (Al-Zamil et al., 2024) [15] utilized a robust, parallel-group randomized controlled trial (RCT) design, providing an adequate active comparator (intramuscular LTPI) and a non-interventional control group. However, even within this high-quality trial, patient and clinician blinding was methodologically unfeasible due to the nature of the invasive intraosseous puncture, though outcome assessors were blinded to mitigate performance and detection biases. In contrast, the remaining four studies (Kornilova et al., 2023 [86]; Sokov E. et al., 2011 [85]; Sokov et al., 2014 [83]; Sokov et al., 2008 [84]) relied on open-label, non-randomized single-group or two-group pretest–posttest designs. These non-RCT profiles feature inherent limitations regarding allocation concealment and comparator adequacy, as three of these studies lacked parallel control arms entirely. Furthermore, the risk of major confounding interventions (such as prior or concurrent systemic analgesics or physical therapy) cannot be completely excluded in these retrospective and pilot cohorts.

Regarding data integrity and safety tracking, attrition bias appears low across the cohorts, with minimal missing data reported. However, adverse-event ascertainment was primarily limited to passive surveillance of acute peri-procedural complications, rather than structured, long-term active safety monitoring. This limitation is compounded by the fact that three out of the five studies (Sokov E. et al., 2011 [85]; Sokov et al., 2014 [83]; Sokov et al., 2008 [84]) did not assess follow-up outcomes beyond the immediate post-treatment period. Consequently, while the compiled data demonstrate strong immediate analgesic trends, these methodological limitations restrict the ability to draw definitive conclusions regarding long-term safety profiles, selective reporting, or comparative clinical superiority.

#### 4.3.3. Concluding Remarks and Evidentiary Limitations

As a narrative review, this paper synthesizes a highly heterogeneous group of primary clinical studies. It is critical to emphasize that the available evidence from these five papers possesses inherent methodological limitations (detailed in Table 2), including small cohort sizes, a predominance of uncontrolled pre–post designs, and relatively short follow-up periods. Furthermore, robust participant and clinician blinding was methodologically unfeasible due to the invasive nature of the intraosseous intervention. Consequently, while the compiled data provide preliminary signals of potential clinical benefit, they do not constitute definitive evidence of therapeutic superiority or absolute safety. These limitations restrict the immediate generalizability of the findings and underscore the critical need for future well-powered, long-term randomized controlled trials.

### 4.4. The Pharmacological Component of Intraosseous LTPI

The observed therapeutic outcomes of intraosseous LTPI can likely be explained by the specific pathophysiological mechanisms discussed above. Given the pharmacological composition—predominantly consisting of lidocaine combined with a minor dose of dexamethasone—the immediate post-procedural onset of analgesia underscores the dominant role of lidocaine and its direct interaction with intraosseous nociceptors [92,93,94,95]. Nevertheless, the anti-inflammatory role of dexamethasone cannot be discounted, as its therapeutic effect typically manifests 2 to 3 h post-injection. However, as noted previously, such conservative dosing may not fully achieve a sustained therapeutic window. Crucially, the definitive role of the local anesthetic in securing immediate analgesia is further substantiated by the sole available study utilizing lidocaine monotherapy [15].

Concurrently, critical contraindications regarding the corticosteroid adjunct must be acknowledged. The application of dexamethasone is strongly discouraged in patients suffering from diabetes mellitus, uncontrolled arterial hypertension, or an elevated risk of developing avascular bone necrosis. In these specific patient populations, the therapeutic protocol should be carefully tailored to eliminate the corticosteroid component, thereby avoiding systemic metabolic, cardiovascular, or ischemic complications.

### 4.5. The Non-Pharmacological Component of Intraosseous LTPI

It is crucial to emphasize that there are no data in the literature regarding the analgesic and anti-inflammatory efficacy of conventional intraosseous autologous blood therapies. Furthermore, the blood aspiration step—which is a mandatory procedural safety measure to confirm proper needle placement within the cancellous bone—cannot be classified as a form of autologous blood injection. First, this aspirated blood completely unmodified and unaltered is reinjected completely unaltered in terms of its immunological status. Second, it is returned within seconds directly into the exact same cancellous bone from which it was drawn.

Another critical pathogenetic mechanism proposed in the development of vertebrogenic pain is the elevation of intraosseous pressure within the spinous processes and adjacent bone structures sharing the same segmental innervation. To date, the exact degree of intraosseous pressure reduction achieved via the cortical puncture performed during intraosseous LTPI remains unexamined. However, by drawing a cautious analogy to multiple small-diameter drilling decompression techniques, it is hypothesized that this repetitive small-diameter puncture may facilitate mechanical decompression, thereby potentially reducing intramedullary hypertension and subsequently decreasing the irritation of intraosseous nociceptors, which ultimately mitigates the pain syndrome.

It is suggested that due to the minimal diameter of the puncture channels, this proposed decompressive effect is not expected to manifest immediately, but rather develops gradually over several hours. Concurrently, this repetitive small-diameter puncture triggers localized reparative osteogenesis, eventually leading to the ossification and subsequent closure of these channels. This delayed mechanical onset further underscores the predominant role of lidocaine in achieving the immediate, post-procedural analgesic effect observed in clinical practice. Nevertheless, further primary research is strictly required in this direction, specifically involving direct intraosseous pressure monitoring before and after cancellous space access, without the confounding administration of any pharmacological agents.

### 4.6. Intraosseous LTPI Targeted into the PSIS Trigger Points

Particular attention should be paid to the analgesic effect achieved by intraosseous LTPI targeted into PSIS. In the reviewed literature, a detailed evaluation of intraosseous LTPI application to this osseous trigger point was exclusively presented in the study by Al-Zamil (2024) [15], where the therapeutic efficacy of this approach was demonstrated to be superior to interventions targeting the L5 and S1 spinous processes.

Although the initial description of intraosseous LTPI in the PSIS dates back to 1988 [109], several controversial questions have since emerged regarding the exact source of this osseous trigger point and its relationship with possible coexisting pathologies within the pelvic region. Since then, clinical examination of patients with vertebrogenic LBP has involved a thorough screening process utilizing both targeted physical examination and advanced biochemical and imaging diagnostic modalities to rule out underlying pathologies of the pelvic bones, as well as the sacroiliac and hip joints.

Importantly, a trigger point in nature is distinguished from a simple pain point by the fact that localized pain and motor responses in trigger points are primarily evoked upon deep palpation and percussion, whereas most patients may not present with spontaneous complaints regarding this localized tenderness.

In the study of Al-Zamil 2024 [15], a rigorous differential diagnosis was performed to exclude other potential drivers of low back pain. The inclusion criteria were strictly restricted to patients presenting with lumbosacral pain, while the exclusion criteria explicitly ruled out individuals with underlying rheumatic conditions or signs of ankylosing spondylitis. To achieve this, in accordance with international guidelines, a comprehensive rheumatology panel including HLA-B27 testing and magnetic resonance imaging (MRI) of the sacroiliac and hip joints was performed. Consequently, this study could not have included patients with concomitant pelvic bone pathology, hip osteoarthritis, or sacroiliac joint dysfunction, which could have accounted for the presence of trigger points in the PSIS region. In our opinion, this local tenderness may be classified as referred pain resulting from segmental sensitization. Mechanistically, this phenomenon is tightly analogous to the formation of secondary myofascial trigger points in the gluteal or piriformis muscles, which commonly develop in response to chronic vertebrogenic nociception from the same spinal cord segments. However, it is methodologically difficult to completely eliminate concurrent subclinical sources of pain, such as localized mechanical sacroiliac joint pain, posterior ligamentous or entheseal disorders, superior cluneal nerve pathology, myofascial pain, or imaging-negative referred pain.

In conclusion, given that this is the only study to perform such a comparison and considering its small sample size, the clinical advantages of blocking this specific trigger point cannot be definitively established. Further studies with larger cohorts and more detailed diagnostic examinations are required to clarify the nature of histological and biochemical changes in the PSIS region, even in the absence of MRI-detected pathologies.

### 4.7. Short-Term Analgesic Effect

It is essential to emphasize that all available studies evaluating intraosseous LTPI were conducted on patients presenting with severe pain syndromes. The primary objective of these interventions is to rapidly alleviate acute pain, thereby creating a therapeutic window to proceed with supplementary modalities, particularly non-pharmacological therapies. Nevertheless, a short-term analgesic effect was documented in two of the reviewed trials. This extended efficacy is most likely mediated by the suppression of both central and segmental sensitization processes, which prevents the pain syndrome from returning to its original baseline level driven by underlying spinal degenerative alterations.

### 4.8. Comparison of Intraosseous LTPI with Other Intraosseous Treatment Modalities

To establish the precise clinical and pathophysiological positioning of intraosseous LTPI, it must be compared with other bone-targeted interventions, specifically standalone intraosseous decompression and high-frequency basivertebral nerve (BVN) ablation.

Although these treatment modalities are conceptually unified by their shared objective—either directly or indirectly reducing hyper-afferentation via intraosseous nociceptive pathways—they diverge fundamentally in their underlying mechanisms of action and practical application. First, it must be clarified that isolated intraosseous decompression is not clinically utilized as a standalone therapy for vertebrogenic pain management, rendering a direct practical comparison unfeasible. Instead, high-frequency BVN ablation has recently gained widespread adoption in contemporary spine care [110,111].

As an established intervention, BVN ablation offers the clear advantage of prolonged clinical efficacy validated by multiple large-scale randomized controlled trials. Nevertheless, it may be hypothesized that in specific clinical scenarios where a patient’s severe pain syndrome is driven primarily by a transient amplification of central and segmental sensitization loops, temporary pharmacological and pressure modification via intraosseous LTPI could potentially interrupt the pathological cascade. From a conceptual standpoint, in such cases, temporary neuromodulation might provide short-term relief without requiring immediate irreversible thermal destruction of neural architecture.

However, in the absolute absence of head-to-head comparative trials, intraosseous LTPI cannot be characterized as an equivalent alternative or a direct replacement for BVN ablation. While these interventions share an underlying conceptual target related to bone-derived nociception, their selection protocols, evidence base, invasiveness, and long-term durability remain substantially different, and LTPI should currently be viewed strictly as a preliminary, less destructive option requiring further high-tier validation.

### 4.9. Methodological Challenges in Isolating Multi-Component Mechanistic Pathway

Another critical interpretive challenge arises from the multi-component nature of the evaluated intervention. Because the primary clinical protocols simultaneously integrated mechanical cortical puncture, bone marrow aspiration, and the pharmacological co-administration of lidocaine and dexamethasone, the existing literature does not experimentally isolate the specific therapeutic contribution of each individual element. While the immediate post-procedural analgesia observed across the cohorts is highly compatible with the acute localized anesthetic action of lidocaine, available data are insufficient to firmly establish it as the dominant analgesic mechanism. Similarly, secondary physiological cascades—including decompressive, sympatholytic, vascular, and metabolic pathways—must be treated with methodological caution. In the absence of direct, real-time intraosseous human data, these interconnected cascades should be consistently interpreted as biologically plausible and hypothetical frameworks rather than definitively verified mechanisms.

## 5. Limitations

A primary limitation of this study stems from the distinct scarcity of contemporary global literature on the subject, which necessitated reliance on a limited number of experimental designs and several studies featuring significant chronological obsolescence. Specifically, when assessing the clinical applications of intraosseous LTPI for vertebrogenic pain management, our analysis was constrained to draw heavily upon three foundational, earlier publications.

Additionally, another methodological limitation lies in the demographic homogeneity of the study population, as all enrolled patients across these trials were of European descent. This geographic and ethnic restriction narrows the immediate clinical generalizability of the synthesized outcomes to more diverse global populations, thereby emphasizing the clear necessity for future multi-center, trans-ethnic validation studies.

Another critical limitation of our study is the profound clinical and methodological heterogeneity among the available studies—including the integration of trials entirely lacking long-term follow-up assessments—which is directly attributed to the extreme scarcity of publications that fully meet the rigorous eligibility criteria required for standardized data extraction.

## 6. Conclusions

Age-related degenerative changes within the vertebral bone include hyperactive osteoclastogenesis, intraosseous hypertension, and localized upregulation of neuroinflammatory cytokines and the NGF/TrkA axis, which collectively excite and sensitize intraosseous nociceptive and sensory receptors. It is suggested that the synergistic nociceptive and small-fiber hyperafferentation leads to the development of segmental spinal and central sensitization. However, the pathogenetic link between central sensitization and trigger point formation remains poorly understood. The available clinical studies investigating intraosseous LTPI for vertebrogenic pain are scarce and heterogeneous. The compiled evidence provides preliminary signals of potential benefit, observing a statistically significant analgesic effect of intraosseous LTPI within the analyzed cohorts. Furthermore, promising short-term outcomes were observed compared to intramuscular LTPI in a randomized trial. However, these trials are limited by small sample sizes, methodological heterogeneity, and a lack of long-term follow-up. Nevertheless, although current data provide promising but still limited insights with no registered peri-procedural complications, future double-blinded, multicenter randomized controlled trials are required to comprehensively establish the clinical efficacy, safety profile, and prolonged effect of intraosseous LTPI in the treatment of vertebrogenic pain.

## Figures and Tables

**Table 1:** Summary of study designs, treatment protocols, and clinical outcomes of extracted publications devoted to intraosseous lidocaine trigger point injection in the treatment of musculoskeletal pain.

Au. Years	Study Design	Zone	Sample Size	Interventions	Outcomes	Follow-Up
Kornilova et al. (2023) [86]	Single -Group Pretest–Posttest Design *	Low back pain	Intraosseous LTPI group (n = 13)	2 mL of 2% lidocaine with 2 mg of dexamethasone into the spinous processes of L5, and S1 and the posterior superior iliac spine (3 procedures, once every 5–7 days.)	Before treatment: VAS: 7.0 ± 0.2, MPQ PRI (total): 26.1 ± 2.1, Pain drawing (extent): 2.5% ± 0.6%.), ODI: 33.6 ± 2.3. Thomayer’s test: 19.7 ± 0.7 cm, SLR: 48.0 ± 5.0°, TPC: 7.9 ± 1.3. After treatment: VAS pain: ↓ 44.3% MPQ (total): ↓ 43.6%, Pain area: ↓ 32.0%. ODI: ↓ 40.2%. Thomayer’s test: ↓ 57.9%. SLR: ↑ 46.9%. TPC: ↓ 59.5%.	At 2 months: 100.0% of patients with no negative dynamics. At 3–6 months: 53.8% of patients with no negative dynamics
Sokov E. et al. 2011 [85]	Two-Group Pretest–Posttest Design.	Cervicobrachialgia	Intraosseous LTPI group: (n = 70). Intramuscular LTPI Group: Paravertebral blockade (n = 40)	8 mL of 1% lidocaine with 1 mg of dexamethasone into the spinous processes of C2, C6, C7, T1 vertebrae and scapular spines (5 procedures, every other day).	Before Treatment: Intramuscular LTPI: Shoulder ROM restriction: 35.0°; VAS Pain Score: 5.9 ± 0.2; McGill PRI (Sensory): 17.6 ± 2.0; McGill PRI (Affective): 6.1 ± 0.7. Intraosseous LTPI: Shoulder ROM restriction: 30.0°; VAS Pain Score: 6.0 ± 0.1; McGill PRI (Sensory): 16.1 ± 0.9; McGill PRI (Affective): 5.8 ± 0.6. Post-Treatment Superiority of intraosseous LTPI: Shoulder ROM restriction: ↑ 66.1%. VAS pain: ↓ 43.1% MPQ (sensory): ↓ 27.1% MPQ (affective): ↓ 9.7%	Follow-up outcomes were not assessed.
Sokov et al. 2014 [83]	Single-group Pre-test–Posttest Design.	Cervicalgia	50 (F: 29, M: 21). 21–60 years	8 mL of 1% lidocaine with 1 mg of dexamethasone into the spinous processes of C2, C6, C7, T1 vertebrae and scapular spines (5 procedures, every other day).	Before treatment: VAS Pain Score: 6.2 ± 0.2, McGill PRI (Sensory): 16.2 ± 1.2, McGill PRI (Affective): 8.2 ± 0.5, BDI: 14.4 ± 3.4, STAI-S: 50.3 ± 1.7 After treatment: VAS pain: ↓ 59.7% MPQ (sensory): ↓ 48.1% MPQ (affective): ↓ 70% BDI: ↓ 35.4% STAI-S: ↓ 13.9%	Follow-up outcomes were not assessed.
Al-Zamil M. et al. 2024 [15]	RCT	Residual Chronic Pain after DLSS Decompression Surgery	Total N = 110 (5 parallel arms: Control, n = 21; Intramuscular L4–S1, n = 22; Intramuscular PSIS, n = 22; Intraosseous L5/S1, n = 22; Intraosseous PSIS, n = 23)	All five groups received identical standard background pharmacotherapy. Group 1: Pharmacotherapy only. Groups 2 & 3: Pharmacotherapy + intramuscular LTPI (L4–S1 or PSIS). Groups 4 & 5: Pharmacotherapy + intraosseous LTPI (L5/S1 or PSIS; 4 mL of 2% lidocaine mixed with 2 mL of autologous bone marrow blood aspirate). 5 procedures every 3 days.	Before treatment: VAS: 7.2 ± 0.11 points, tactile hypoesthesia 2.1 ± 0.08/5 points, symptom severity (ZCQ) = 3.42 ± 0.12 points, functional disability (ZCQ) = 2.56. ± 0.11 points. After treatment: Compared to initial values: VAS pain: ↓ 76.9% Tactile hypoesthesia ↓51.7%, Symptom severity (ZCQ): ↓ 44.1%. Functional disability (ZCQ): ↓ 52.9% Compared to IM LTPI: VAS pain: ↓ 57.1% Tactile hypoesthesia ↓ 26.3%, Symptom severity (ZCQ): ↓ 31.4%. Functional disability (ZCQ): ↓ 41.4%	At 4 months follow-up: Intraosseous LTPI: Compared to initial values: VAS Pain: ↓ 38.4% Tactile recovery: ↑ 32.5%. Symptom severity (ZCQ): ↓ 33.3%. Functional disability (ZCQ): ↓ 37.7% IM LTPI: Compared to initial values: VAS Pain: ↓ 27.7% No significant changes in tactile recovery, symptom severity (ZCQ) and functional disability (ZCQ).
Sokov et al. 2008 [84]	Single-group Pre-test–Posttest Design.	Lumbar Failed Back Surgery Syndrome	44 (F: 20, M: 24). 51.9 ± 10.8 years	8 mL of 1% lidocaine with 1 mg of dexamethasone into the spinous processes of L4, L5, and S1, the posterior superior iliac spine (5 procedures, every other day).	Before treatment: VAS: 7.0 ± 1.2, MPQ PRI (sensory): 18.2 ± 9.9, MPQ PRI (affective): 6.7 ± 3.7, Pain drawing (extent): 7.1% ± 2.5%. After treatment: VAS pain: ↓ 45.7% MPQ PRI (sensory): ↓ 43.4%, MPQ PRI (affective): ↓ 38.9% Pain area: ↓ 87.3%.	Follow-up outcomes were not assessed.

Notes: IM—Intramuscular; VAS, visual analogue scale; L4, processus spinosus of the 4th lumbar vertebra; L5, processus spinosus of the 5th lumbar vertebra; S1, processus spinosus of the 1st sacral vertebra; C2, processus spinosus of the 2nd cervical vertebra; C6, processus spinosus of the 6th cervical vertebra; C7, processus spinosus of the 7th cervical vertebra; Th1, processus spinosus of the 1st thoracic vertebra; MPQ, McGill Pain Questionnaire; PRI, pain rating index; ROM, range of motion; SLR, straight leg raise; TPC, trigger point count; ODI, Oswestry Disability Index; ZCQ, Zurich Claudication Questionnaire; LTPI, lidocaine trigger point injection; ↓—Decrease; ↑—Increase; *—The primary study compared intraosseous LTPI against a combined protocol with HYALREPAIR-02 hyaluronic-acid bioreparant. For the specific scope of this review, clinical data were extracted exclusively for the isolated active lidocaine/dexamethasone control cohort (n = 13). Due to the absence of a relevant concurrent control group for head-to-head comparison within this specific extraction baseline, this cohort was methodologically classified as a single-group pretest–posttest profile.

**Table 2:** Methodological quality appraisal and study-level limitations of the evaluated clinical publications.

Au. Years	Control/Comparator Adequacy	Blinding Profile	Allocation Concealment	Attrition & Missing Data	Selective Reporting Risk	Adverse-Event Ascertainment	Major Confounding Interventions
Kornilova et al. (2023) [86]	Inadequate (No concurrent control or non-LTPI arm; evaluated as a single group).	Open-label for patients and clinicians.	Not applicable (Single-group longitudinal design).	Low risk (No dropouts or missing data reported).	Low risk (All clinical outcomes reported).	Passive surveillance (Limited to acute peri-procedural tracking).	Low risk (Evaluated strictly as a monotherapy; concurrent systemic analgesics were excluded by protocol).
Sokov E. et al. (2011) [85]	Adequacy met (Compared head-to-head with paravertebral intramuscular LTPI).	Open-label for patients and clinicians.	Randomized parallel-group allocation.	Low risk (No participant attrition reported).	Low risk (Standard parameters analyzed).	Passive surveillance (No long-term safety protocol).	Moderate risk (All subjects in both arms received identical standard background pharmacotherapy; sample size asymmetry present: 70 vs. 40 patients).
Sokov et al. (2014) [83]	Inadequate (No concurrent control arm; historical/baseline comparison only).	Open-label for patients and clinicians.	Not applicable (Single-group pretest–posttest design).	Low risk (0% dropout rate reported).	Low risk (Psychological scales and pain scores fully reported).	Passive surveillance (Acute complications monitored only).	Low risk (Evaluated strictly as a monotherapy; concurrent systemic analgesics or physical therapies were excluded by study protocol).
Al-Zamil M. et al. (2024) [15]	Highly adequate (Robust 5-arm parallel group add-on design featuring medication control and specific active arms).	Blinded assessors; open-label for patients/operators due to procedural nature.	Randomized (Computer-generated sequence utilized.	Low risk (<2% missing data; all allocated subjects analyzed).	Low risk (All primary parameters mapped to protocols).	Passive surveillance (No long-term active post-discharge protocol).	Low risk (Rigorous add-on design; all 5 arms received identical standard background pharmacotherapy, perfectly isolating the intervention effect).
Sokov et al. (2008) [84]	Inadequate (No concurrent control arm; pilot longitudinal data only).	Open-label for patients and clinicians.	Not applicable (Single-group design).	Low risk (No missing data trends observed).	Low risk (Pain drawings and scales fully reported).	Passive surveillance (Limited to immediate post-treatment period).	Low risk (Evaluated strictly as a monotherapy; concurrent systemic analgesics were excluded, though surgical history heterogeneity remains).

## Data Availability

No new data were created or analyzed in this study. Data sharing is not applicable to this article.

## Supplementary Materials

The following supporting information can be downloaded at: https://www.mdpi.com/article/10.3390/ijms27198935/s1.
