# Supplementary material for: Intraosseous Lidocaine Trigger Point Injection for Vertebrogenic Pain: From Molecular Principles to Clinical Applications: A Narrative Review
Source: Int J Mol Sci. 2026 Oct 8;27(19):8935. doi: 10.3390/ijms27198935 (PMC13665498; doi:10.3390/ijms27198935)
Supplement: Supplementary file 1 [file ijms-27-08935-s001.zip › ijms-4520286-supplementary.pdf]

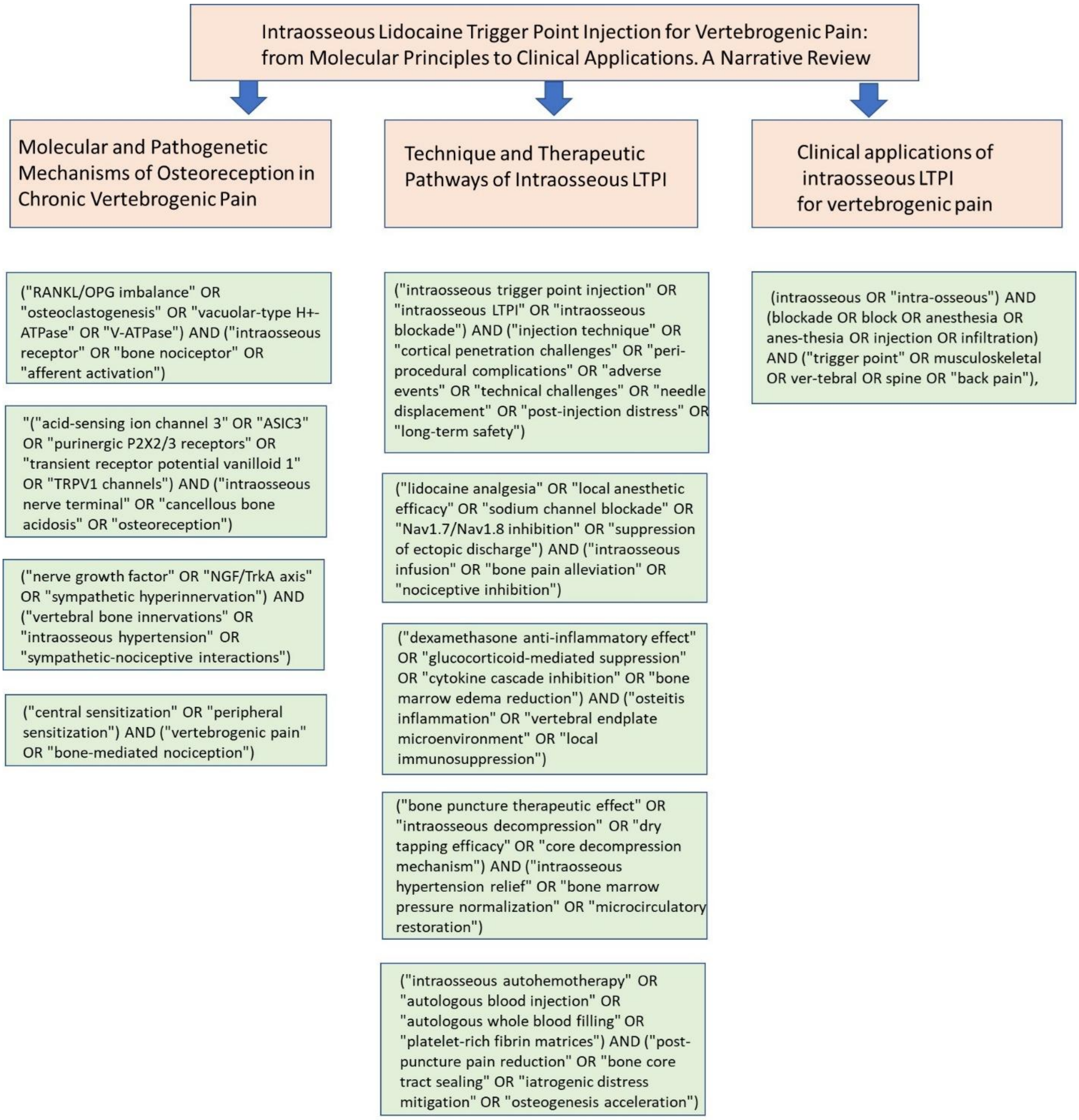

Figure S1. Search strategies and keywords used for the literature search.
